# Isolation of Oct4-Expressing Extraembryonic Endoderm Precursor Cell Lines

**DOI:** 10.1371/journal.pone.0007216

**Published:** 2009-09-28

**Authors:** Bisrat G. Debeb, Vasiliy Galat, Jessica Epple-Farmer, Steve Iannaccone, Wendy A. Woodward, Michael Bader, Philip Iannaccone, Bert Binas

**Affiliations:** 1 Department of Pathobiology, College of Veterinary Medicine and Biomedical Sciences, Texas A&M University, College Station, Texas, United States of America; 2 Developmental Biology Program, Children's Memorial Research Center, Northwestern University, Chicago, Illinois, United States of America; 3 Division of Radiation Oncology, University of Texas M.D. Anderson Cancer Center, Houston, Texas, United States of America; 4 Max Delbruck Center for Molecular Medicine, Berlin, Germany; INSERM, France

## Abstract

**Background:**

The extraembryonic endoderm (ExEn) defines the yolk sac, a set of membranes that provide essential support for mammalian embryos. Recent findings suggest that the committed ExEn precursor is present already in the embryonic Inner Cell Mass (ICM) as a group of cells that intermingles with the closely related epiblast precursor. All ICM cells contain Oct4, a key transcription factor that is first expressed at the morula stage. In vitro, the epiblast precursor is most closely represented by the well-characterized embryonic stem (ES) cell lines that maintain the expression of Oct4, but analogous ExEn precursor cell lines are not known and it is unclear if they would express Oct4.

**Methodology/Principal Findings:**

Here we report the isolation and characterization of permanently proliferating Oct4-expressing rat cell lines (“XEN-P cell lines”), which closely resemble the ExEn precursor. We isolated the XEN-P cell lines from blastocysts and characterized them by plating and gene expression assays as well as by injection into embryos. Like ES cells, the XEN-P cells express Oct4 and SSEA1 at high levels and their growth is stimulated by leukemia inhibitory factor, but instead of the epiblast determinant Nanog, they express the ExEn determinants Gata6 and Gata4. Further, they lack markers characteristic of the more differentiated primitive/visceral and parietal ExEn stages, but exclusively differentiate into these stages in vitro and contribute to them in vivo.

**Conclusions/Significance:**

Our findings (i) suggest strongly that the ExEn precursor is a self-renewable entity, (ii) indicate that active Oct4 gene expression (transcription plus translation) is part of its molecular identity, and (iii) provide an in vitro model of early ExEn differentiation.

## Introduction

Before implanting into the uterine wall, the mammalian conceptus specifies the cell types that are the founders of trophoblast, extraembryonic endoderm, and fetus. The first morphologically distinct cell type of the trophoblast lineage is the trophectoderm, which becomes discernible at the morula stage and gives rise to the placental trophoblast. The first morphologically distinct cell type of the extraembryonic endoderm is the primitive endoderm, which at the late blastocyst stage becomes visible as a cell layer on the mural surface of the Inner Cell Mass (ICM) and gives rise to the yolk sac endoderm with its visceral and parietal components. Finally, the first morphologically distinct cell type of the fetal lineage is the epiblast, which constitutes the remainder of the late ICM and gives rise to amnion, extraembryonic mesoderm, and embryo proper [1].

Cultured cell lines that maintain or acquire pre- or peri-implantation embryo cell type identities offer great promises for biotechnology and medicine. Prototypical of such cell lines are the well-known mouse embryonic stem (ES) cells [2], [3], which closely resemble the nascent epiblast [4]. ES cells have also been recently isolated in the rat [5], [6], and similar human cells appear to exist as well [7]. In addition, rat and mouse stem cell lines that closely resemble the post-implantation epiblast have been isolated and were found to have gene expression profiles and transcription factor networks similar to the well-known human “ES cells” [8], [9]. Thus, cell lines that can represent the earliest stages of the fetal pathway in vitro exist and appear to be remarkably similar across mammalian species.

The situation is less clear regarding cell lines representing the trophoblast and extraembryonic endoderm lineages. Trophoblast stem (TS) cell lines have been isolated from blastocysts in the mouse [10] and apparently rat [11], but have not yet been reported from humans. Other cell lines with trophoblastic (and perhaps extraembryonic-endodermal) differentiation potential [11]–[13] have also been derived from rat blastocysts, but remain poorly characterized and of uncertain in vivo potential. Furthermore, extraembryonic endoderm stem cell lines called “XEN cells” (“XEN” for extraembryonic endoderm) have been isolated from mouse blastocysts [14]. These XEN cells can efficiently contribute to parietal endoderm in vivo, but they did not efficiently integrate into the visceral endoderm. Therefore, they may not represent the first committed step of the extraembryonic endoderm (i.e., the committed extraembryonic endoderm precursor). It may be significant in this context that XEN cells do not express the transcription factor Oct4 [14] that is found in all cells of the early ICM [15]. Indeed, a recent analysis of mouse blastocysts has raised the possibility that the committed extraembryonic endoderm precursor exists already in the early ICM [16], [17], although the status of *Oct4* gene transcription in these putative extraembryonic endoderm precursor cells is not clear.

Here we show that from rat blastocysts, cell lines with extraembryonic endoderm identity can be derived that are distinguished from XEN cells by a less mature marker spectrum (including Oct4) and a better ability to form visceral endoderm (in addition to parietal) in vitro and in vivo. These cells appear to represent the first committed step of the extraembryonic endoderm lineage, and we therefore name them XEN-P cells (“P” for precursor).

## Results

### Generation of rat cell lines that express both ICM and extraembryonic endoderm markers

**Figure 1:**
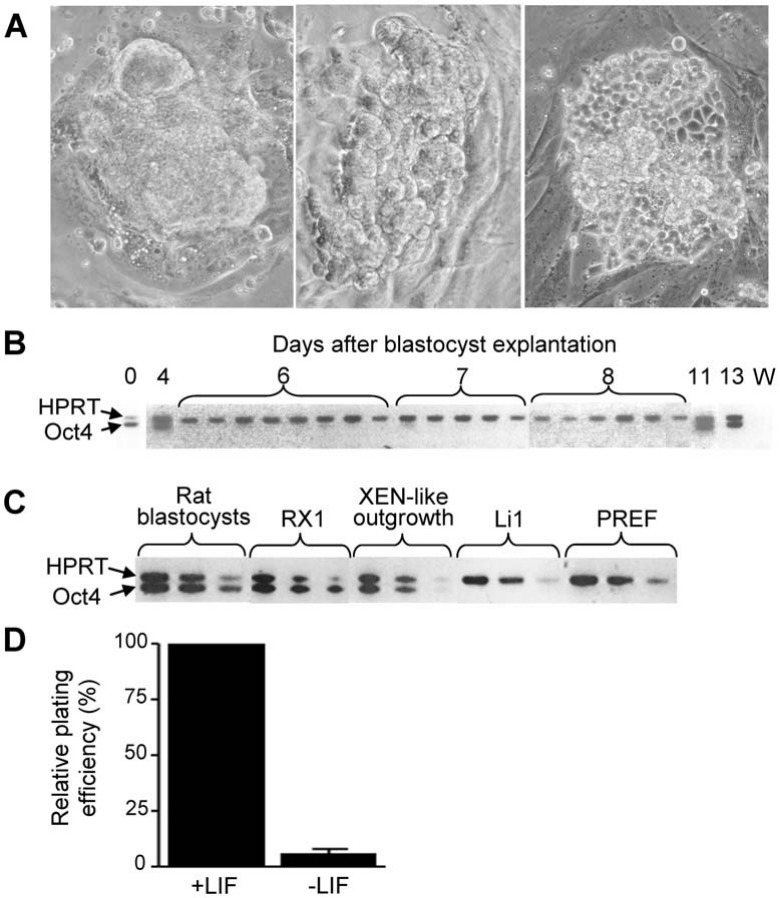
Properties of rat blastocyst outgrowths. (A) Phase contrast photographs showing stages of WKY rat blastocyst outgrowths kept on mitomycin-treated primary rat embryo fibroblasts (PREF). The outgrowths were initially smooth and compact (left), but converted to XEN morphology (right) ∼10 days after blastocyst plating if not passaged, or a few days later if mechanically disaggregated into smaller clumps. Regardless of when the conversion occurred, it was fast (<24 hours) and went through a stage of intermediate morphology (middle). (B) Loss and re-expression of Oct4 mRNA in WKY rat blastocyst outgrowths. In these experiments, the outgrowths were not passaged and showed compact, smooth morphology before day 10, but XEN morphology thereafter. At the indicated days, the outgrowths were individually harvested for RT-PCR analysis, using rat-specific primers for Oct4 and hypoxanthine phosphoribosyl transferase (Hprt) cDNAs. The Oct4 and Hprt cDNAs were amplified in the same reaction; none of the primers amplified intronless products from genomic DNA (not shown). No amplification was achieved when using mouse-specific primers (not shown). Day 0 = blastocyst; W, water control. (C) Semi-quantitative assessment of Oct4 mRNA level. Rat blastocysts (E4.5, strain WKY), XEN-P line RX1, primary XEN-like blastocyst outgrowths (strain WKY), rat embryo fibroblast line Li 1 (feeder for RX1), and PREF (feeder for primary rat cells) were analyzed for Oct4 and Hprt mRNAs by subjecting 10-fold serial dilutions of the RT reactions to PCR. (D) LIF effect (1,000 u/ml) on the formation of secondary XEN-like cell colonies from primary rat blastocyst outgrowths (WKY). Primary cells were seeded at ∼100–500 cells/well onto feeder line Li 1. 6 independent experiments. Similar results were obtained with rat strain BDIX.

When explanted onto mitotically inactivated primary embryo fibroblasts, rat blastocysts produced smooth, compact outgrowths that initially grew rapidly and could be passaged a few times. After 10–20 days, however, these outgrowths usually converted abruptly into cells with a morphology similar to that of earlier published rodent extraembryonic endoderm cell lines [14], [18] that, in the mouse, were termed XEN cell lines [14] (Fig. 1A). Strikingly, this conversion was associated with the re-expression of Oct4 mRNA that had been lost after day 4 (Fig. 1B, C). At low density, the primary rat blastocyst-derived cells formed colonies with a morphology that was also XEN-like, and this colony formation was stimulated by leukemia inhibitory factor (LIF) (Fig. 1D). Both primary rat embryo fibroblasts and a rat embryo-derived permanent fibroblast cell line (Li1) derived in our laboratory were suitable as feeder cells, but mouse embryo fibroblasts produced extremely variable (batch-dependent) results. When the feeder cells were omitted, colony formation at low density was reduced to an insignificant fraction (on plastic) or undetectable (on gelatin-coated plastic).

**Figure 2:**
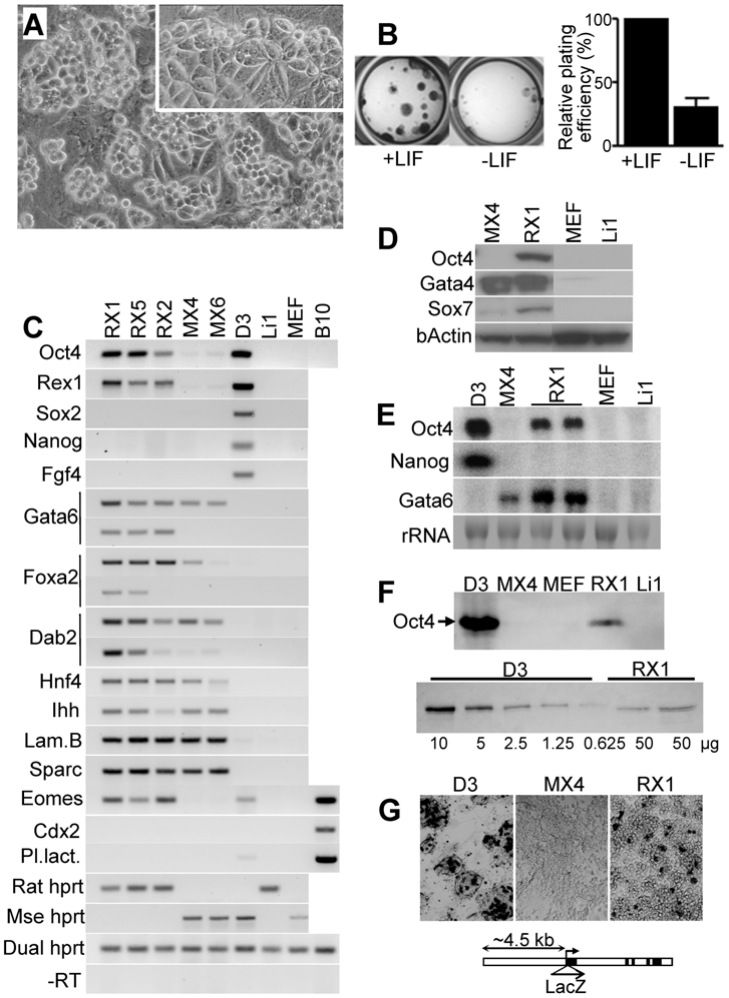
Growth behavior and comparative embryonic lineage marker analysis of rat XEN-P cell lines. (A) Phase contrast photo showing characteristic morphology of rat XEN-P cell lines growing on rat embryo fibroblast feeder. Colonies obtained by low-density plating typically contained round, refractile cells at their fringes and epithelial cells inside (inset). (B) Representative photos illustrating that LIF (1000 u/ml) increased colony diameter and frequency (crystal violet staining) (line RX1). Similar results were obtained with line RX2 (strain BDIX). (C) RT-PCR analysis showing that rat XEN-P cell lines exhibit a mixed embryonic lineage marker profile. Rat XEN-P cell lines (RX1, RX2, RX5) were compared with mouse XEN cell lines (MX4, MX6), a mouse ES cell line (D3), a trophectoderm-like rat cell line (B10), a rat embryo fibroblast cell line (Li1) used as feeder for the XEN-P cell lines, and primary mouse embryo fibroblasts (MEF) used as feeders for mouse XEN and ES cells. Lines D3 and B10 have been described before [43], [13]. 2 µg of RNA per sample were reverse-transcribed or not (-RT), followed by PCR using dual-specific (rat = mouse) primers. For Gata6, Foxa2, and Dab2, two dilutions of the RT reaction were subjected to PCR for semi-quantitative comparison. (D) Western blot analysis of XEN-P (RX1), mouse XEN (MX4), and feeder (MEF, Li1) cell lines. 40 µg of cell protein were loaded per lane. (E) Northern blot analysis of XEN-P (RX1), mouse XEN (MX4), mouse ES (D3), and feeder (MEF, Li1) cell lines. 5 µg of total RNA were loaded per lane. (F) Western blot analysis for Oct4 in rat XEN-P (RX1), mouse XEN (MX4), mouse ES (D3), and feeder (MEF, Li1) cell lines, using a monoclonal anti-Oct4 antibody. 50 µg (top) or the indicated amounts (bottom) of cell protein were loaded. RX1 samples from two passages (P39, P40) were analyzed (bottom). Similar results were obtained with a polyclonal antibody (not shown). (G) Transient expression of mouse Oct4 gene-based LacZ reporter gene GOF9 [53] by rat XEN-P and mouse ES but not mouse XEN cell lines. Histochemical stainings of lines D3, MX4, and RX1 (similar results were obtained with line RX2). Non-transfected cells did not show LacZ staining (not shown). When comparing the frequencies of reporter gene expression in mouse ES vs. rat XEN-P cell lines, keep in mind that only a subpopulation in the rat cell lines highly expresses the endogenous *Oct4* gene (Fig. 3).

**Figure 3:**
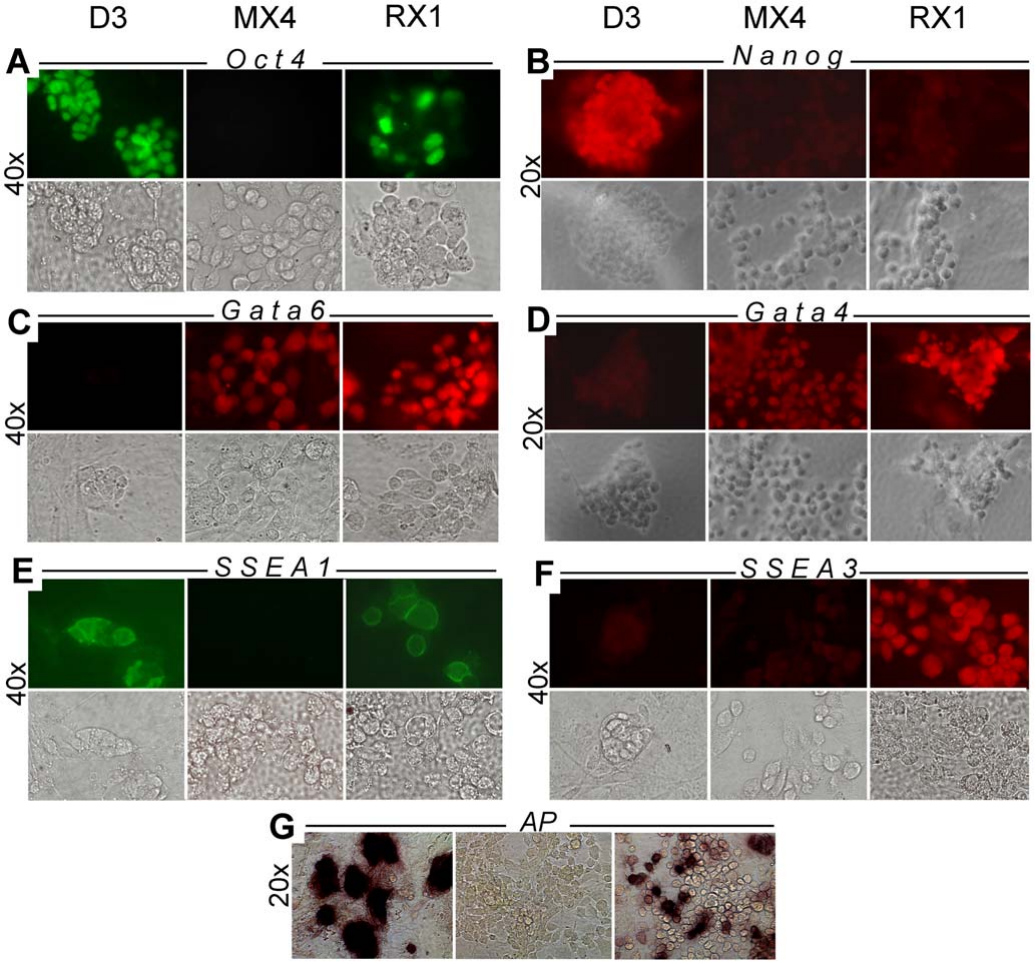
Heterogeneity of lineage marker expression in rat XEN-P cell lines. Rat XEN-P (line RX1), mouse XEN (line MX4), and mouse ES (line D3) cell lines were analyzed by indirect immunofluorescence with antibodies specifically recognizing Oct4 (A), Nanog (B), Gata6 (C), Gata4 (D), SSEA1 (E), or SSEA3 (F), or they were stained for alkaline phosphatase activity (G). Controls in which primary antibodies were omitted were negative and are not shown. Upper rows show immunofluorescence, lower rows show bright field images. Using immunocytochemistry, we also analyzed Oct4 and SSEA1 expression in line RX2 and SSEA1 expression in line RX5; the lines were positive, but the percentages of positive cells were lower than in line RX1 (results not shown).

Intrigued by the Oct4 expression, we established three independent cell lines (RX1, RX5 - strain WKY; RX2 –strain BDIX) by transferring small pools of XEN-like blastocyst outgrowths onto Li1 feeders; the cells simply kept growing, and no “crisis” was noticed. Since derivation was so easy, no attempt was made to increase or exactly quantify the efficiency. The resulting cell lines maintained the XEN-like morphology (Fig. 2A) and Oct4 mRNA expression (Fig. 1C, Fig. 2C) of the primary cells. Lines RX1 and RX2 were also tested for their LIF responsiveness, which was maintained (Fig. 2B), and line RX1 was further tested on different supports and found to maintain the differential behavior on feeder, plastic, and gelatin seen with the primary cells. We designate these cell lines, which are routinely maintained on mitotically inactivated feeders (cell line Li1), as XEN-P cell lines and arbitrarily chose line RX1 for the majority of analyses described below. In these cells, sizes of the Oct4 mRNA and of the resulting protein corresponded to those in mouse ES cells (Fig. 2E, F), indicating that the XEN-P cell lines expressed the true *Oct4* gene. In agreement with this, transiently transfected lines RX1 and RX2 expressed a reporter gene driven by the regulatory sequences of the mouse *Oct4* gene (Fig. 2G). By contrast, only a faint band was seen after RT-PCR for Oct4 in mouse XEN cells (Fig. 2C), but these mRNA levels were negligible when quantified by real-time qRT-PCR (Table 1), undetectable by Northern blotting (Fig. 2E), did not result in any measurable Oct4 protein (Fig. 2D,F; Fig. 3A), and no reporter gene expression was detected (Fig. 2G). At the same time, all extraembryonic endoderm markers tested were detected by RT-PCR or Western blotting (Fig. 2C,D) in all rat XEN-P and mouse XEN cell lines, including the pan-extraembryonic endoderm markers Gata6 and Gata4, the parietal endoderm marker Sparc, the primitive and visceral endoderm markers Dab2 and Foxa2, and the transcription factor Sox7 that discriminates extraembryonic from definitive endoderm [19]. However, Gata6, Dab2, Foxa2, and Sox7 were expressed at significantly higher levels in rat XEN-P than mouse XEN cell cultures. In addition, all rat XEN-P but not the mouse XEN cell lines expressed Rex1, an ICM/ES cell/extraembryonic ectoderm marker [20], as well as Eomesodermin, a marker of trophectoderm and anterior visceral endoderm [21]. Importantly, the essential ES cell marker Nanog [22] was undetectable by RT-PCR, Northern blotting, and immunocytochemistry (Fig. 2C,E; Fig. 3B), and so were two less specific ES cell markers, Sox2 and Fgf4 (Fig. 2C). Furthermore, the trophectoderm markers Cdx2 and Placental Lactogen were absent from both rat XEN-P and mouse XEN cell lines. In summary, mouse XEN and rat XEN-P cell lines showed comparable expression of parietal endoderm markers, but the rat XEN-P cell lines showed higher levels of visceral endoderm markers and also expressed early lineage markers not found in the mouse XEN cells. The growth requirements of rat XEN-P and mouse XEN cells differed as well: In several independent plating experiments, XEN cells (line MX4) formed no colonies on Li1 cells that supported XEN-P (line RX1) colony growth, but grew well on primary mouse embryo fibroblast batches that failed to support XEN-P colony formation.

**Table 1:** Quantitative RT-PCR for Oct4 in whole-culture (*) and micro- (**) XEN-P cell line samples.

Cells	Species	Strain	Line	Oct4 mRNA
XEN-P cell lines	Rat	WKY	RX1	A 2.10±0.41** (7)
				**^B^** 0.19±0.05** (3)
				0.84*/0.30* (2)
				**^C^** 0.62*/0.67* (2)
			RX5	0.46* (1)
		BDIX	RX2	0.22±0.1** (4)
XEN cell lines	Mouse	NMRI	MX4	0.003±0.001(4)*
		B6xBalb/c	MX6	0.0003* (2)
ES cell line	Mouse	129	D3	1.00* (reference)

Samples (numbers in brackets) were RNA-extracted, RNA preparations were DNAse-treated and quantified in duplicate by real-time RT-PCR using dual-specific (mouse = rat) primers; controls without reverse transcriptase did not yield a product. Data (Means±SEM) were normalized to Hprt mRNA and expressed as fold of the level in ES cells, i.e. ES cell level is set as 1.

A , ^B^, two groups of microsamples with high and moderate Oct4 mRNA expression, respectively. Two experiments labeled ^C^ were corrected for feeder cell RNA; the other measurements are slight underestimates.

In view of the mixed-stage expression pattern and because the level of Oct4 mRNA/protein was lower in rat XEN-P than ES cell lines (Fig. 2E, F), we suspected that our rat XEN-P cell lines were heterogeneous. When we compared the Oct4 mRNA levels of whole cell cultures and microsamples (<500 cells) from XEN-P lines RX1, RX2, and RX5 with those of mouse ES cells ( = 100%) by qRT-PCR, the Oct4 mRNA contents ranged from 22–210% in the microsamples and from 30–84% in whole cultures (Table 1). Thus, although the Oct4 mRNA level was lower in rat XEN-P cell lines on average, it did reach levels higher than in ES cells in a subpopulation. Using line RX1, we then visualized the heterogeneity by immunocytochemistry and found that typically, ∼5–15% (occasionally up to 25%) of rat cells expressed Oct4 highly while the remaining cells exhibited very low but detectable amounts of Oct4 (Fig. 3A). Similarly, line RX1 expressed the ICM/ES cell markers Alkaline Phosphatase and SSEA1 [23] in a significant minority of the cells, and the primitive/visceral endoderm marker SSEA3 [24] in a clear majority of the cells, while none of these markers was expressed by mouse XEN cells (Fig. 3E–G). In addition, all the rat cells showed at least moderate levels of Gata6 and Gata4 but a significant fraction expressed these proteins at higher levels than mouse XEN cells (Fig. 3C,D).

### In vitro differentiation of XEN-P cells causes cell line heterogeneity

**Figure 4:**
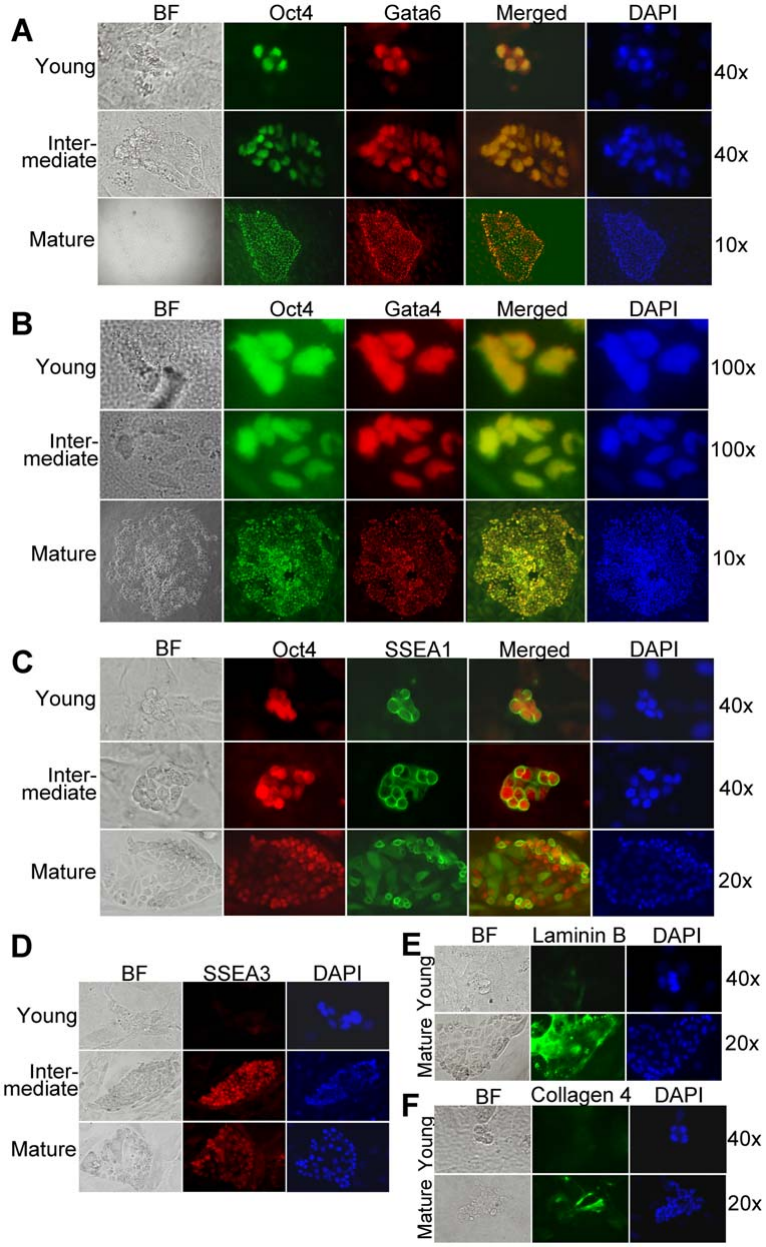
Lineage marker analysis during the evolution of colonies growing after low-density plating of a rat XEN-P cell line (RX1). (A) Double staining for Oct4 (green) and Gata6 (red); (B) Double staining for Oct4 (green) and Gata4 (red); (C) Double staining for Oct4 (red) and SSEA1 (green); (D) Staining for SSEA3; (E) Staining for Laminin B; (F) Staining for Collagen 4. BF, bright field. RX1 cells were plated at 25–50 cells/cm^2^, and at different time points, the resulting colonies were stained with the indicated antibodies and counterstained with DAPI. Controls omitting primary antibodies were negative and are not shown. The speed of colony evolution varied somewhat between experiments, resulting in “Young” colonies at days 2–3, “Intermediate” colonies at days 3–5, and “Mature” colonies at days 5–7 (day 0 = day of plating).

**Figure 5:**
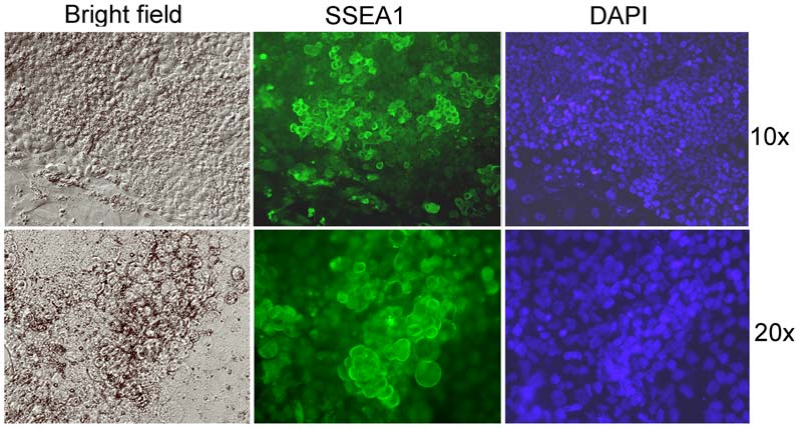
Continued proliferation and preferential accumulation of SSEA1-positive cells in old rat colonies derived from rat XEN-P cells. Two magnifications of a representative 16-days old colony (line RX1) are shown. Bright field (left), immunofluorescence (middle), and nuclear stain (right). Control omitting primary antibody was negative and is not shown.

**Figure 6:**
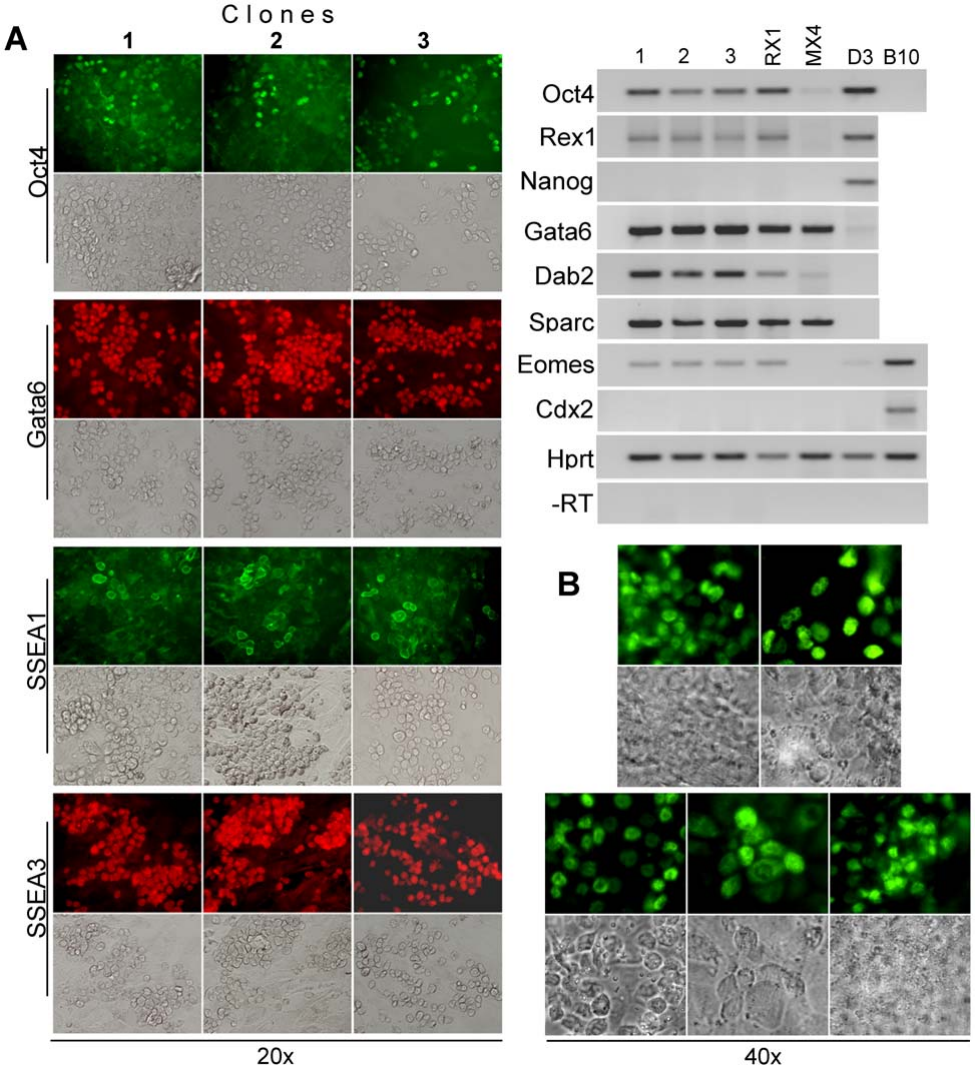
Lineage marker expression in clonal XEN-P cell lines. (A) Line RX1 was subcloned by stable transfection, and three different primary colonies were arbitrarily picked, expanded, and analyzed by indirect immunofluorescence (left; compare to Fig. 3) or RT-PCR (right; compare to Fig. 2C). (B) Line RX1 was subcloned by single-cell FACS deposition. 20 clones were analyzed for Oct4 expression (all positive), and 5 representative photos are shown.

In order to understand the origin of culture heterogeneity and the identity of the self-renewing population, we plated line RX1 at low density and studied the resulting colonies over time. Strikingly, nearly all colonies consisted initially (2–3 days after seeding) entirely or almost entirely of round cells that highly co-expressed Oct4, Gata6, Gata4, and SSEA1 (Fig. 4A–C) while lacking the primitive/visceral endoderm marker SSEA3 as well as the basement membrane components Laminin B and Collagen 4 (Fig. 4D–F) that are characteristically produced by extraembryonic endoderm cells and especially parietal endoderm [25]. In line with the lack of a basement membrane, the young colonies were poorly adherent and easily lost during washing steps. By contrast, in older, larger colonies (4–7 days after seeding), the inner cells became epithelial and firmly adherent, and many round as well as the epithelial cells were negative for SSEA1 and very low in Oct4. Rather, many of the round cells now expressed SSEA3 (Fig. 4D), and the epithelial areas contained abundant, extracellular Laminin B and Collagen 4 (Fig. 4E, F). Notably, however, Oct4/SSEA1-positive cells always persisted in the older colonies, usually at the colony fringes and later also accumulating on top; these same cells tended to show also higher levels of Gata6 (and, to a lesser degree, of Gata4) than the rest of the colony, in line with higher expression in rat XEN-P vs. mouse XEN cell lines (Fig. 2C,E; Fig. 3C, D). With further evolution of the colonies (7–14 days after seeding), the round fringe cells kept proliferating, piled up on top of the colonies (Fig. 5; see also Fig. 4B), and eventually (10–20 days after seeding) converted into bridge-like ductal structures while the inner epithelial parts lost their nuclei and then degenerated completely (results not shown). These data strongly suggest that the round, undifferentiated Oct4/SSEA1-positive cells are the principal self-renewing entity and the precursors of the extraembryonic endoderm cells, i.e. are the XEN-P subpopulation within our XEN-P cell lines. In order to obtain formal evidence that one cell can generate the whole culture heterogeneity, we performed two additional experiments. First, we transfected line RX1 stably with a neomycin resistance marker, a method that ensures single cell origin better than trypsinization. All colonies emerging from G418 selection showed the identical round-epithelial morphology of the parent culture, and three randomly chosen clones showed the same heterogeneous Oct4/Gata6/SSEA1/SSEA3 expression and mixed ExEn lineage marker gene expression as the parent line (Fig. 6A). Second, we sub-cloned line RX1 by single-cell FACS deposition into 96-well plates. Out of 384 wells (in two independent experiments), 129 contained colonies, all of which maintained the heterogeneous morphology of the parent line. 20 colonies were randomly selected for immunostaining, and all contained a significant fraction of Oct4-positive cells (Fig. 6B).

### XEN-P cells contribute to visceral and parietal endoderm in vivo

**Figure 7:**
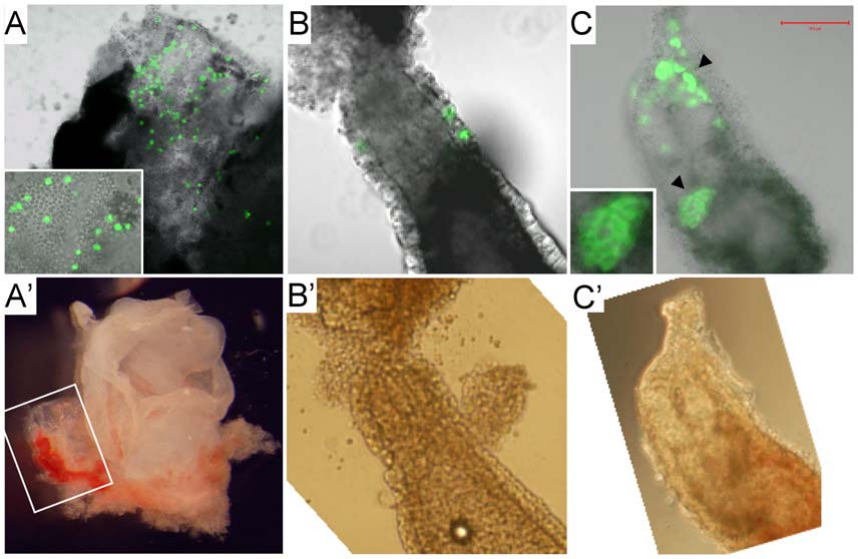
Contributions of rat XEN-P cell lines to postimplantation embryos. Representative fluorescence (A–C) and bright field (A'–C') photographs demonstrating in vivo contributions of microinjected rat cells to (A, A') parietal yolk sac of an 11.5 dpc rat conceptus (inset showing magnification); (B, B') visceral endoderm of an 8.5 dpc rat conceptus; (C, C') visceral endoderm (arrowheads; one patch magnified in inset) of an ∼7 dpc mouse conceptus. Pregnancy timing is distorted by the embryo manipulations and therefore only approximate.

In order to judge their developmental potential, we labeled the XEN-P cell lines with green fluorescent protein (GFP) by lentiviral transduction. Upon injection into rat and mouse blastocysts and subsequent embryo transfer, the labeled cells proliferated and contributed to the parietal (84%) and visceral (12%) layers of rat and mouse yolk sacs (Fig. 7; Table 2). Thus, the cultured rat cells contributed more than sporadically to the visceral endoderm, although they did more often contribute to the parietal endoderm. This preponderance of parietal endoderm integration contrasts with our finding that a majority of cultured rat cells carried the primitive/visceral marker SSEA3 (Figs. 3F, 4D, 6A) but is in line with the preferential contribution of blastocyst-injected primary primitive endoderm and visceral endoderm cells to the parietal endoderm [26]. Also of note, the percentage of visceral endoderm contribution we observed roughly corresponded to the percentage of XEN-P cells in the cultures. Given that all the cell types of the rat cell lines can be derived from a single cell in vitro (Fig. 6), these results imply that the in vivo integrants were at least indirectly derived from cultured XEN-P cells.

**Table 2:** Incorporation of cells rat XEN-P cell lines into postimplantation conceptuses.

No. of Expts.	Cell line	Host species	Injected embryos	Implanta-tion sites	Regressed embryos	Recovery Day	VYS	PYS	Unclear
3	RX1	Rat	30	24	5	8.0–11.5		1	
4	RX5	Rat	59	39	9	8.5–9.5	1	7	
3	RX1	Mouse	34	25	15	7.0–8.0	2	6	1*
4	RX5	Mouse	59	37	20	7.5–8.5		7	
						Sum (%)	3 (12)	21 (84)	1* (4)

A total of 145 rat and 168 mouse embryos were injected and transferred in 13 and 15 experiments, respectively. Included in the table are only those experiments in which labeled cells could be recovered in a structure of the intact conceptus, including visceral yolk sac (VYS) endoderm and parietal yolk sac (PYS) endoderm. (*) Abnormal embryo with a patch of fluorescent cells.

## Discussion

In view of the short-lived, transitory nature of the preimplantation embryo, it is not self-evident that its cells can multiply perpetually without losing identity and that all of their mRNAs and proteins result from actual gene transcription. The existence of immortal ES cell lines, now thought to represent the committed epiblast precursor/naive epiblast [16], [4], is therefore remarkable, and the fact that they express Oct4 is a strong indicator that the *Oct4* gene is actively transcribed at this developmental stage. The data presented here argue that just like the beginning epiblast, the committed extraembryonic endoderm precursor can be cultured permanently in vitro and actively transcribes the *Oct4* gene.

The cell lines that we derived from rat blastocysts contain a subpopulation, which we term XEN-P cells, whose molecular signature can be summarized as Oct4^+^ SSEA1^+^ Gata6^+^ Gata4^+^ Nanog^−^ Cdx2^−^. Of these markers, Oct4 and SSEA1 are characteristic of the morula and the ICM [15], [23], while Gata6 and Gata4 not only identify the committed extraembryonic endoderm precursor [16], [17] but are actually capable of inducing an extraembryonic endoderm identity [27], [28]. Further, absence of Nanog distinguishes the XEN-P cells from the committed epiblast precursor at least as proposed for mouse [16], [17] and from all known ES cells including rat ES cells [5], [6], while lack of Cdx2 distinguishes the XEN-P cells from previously described rat extraembryonic [11], [13] and trophoblast [10] stem cells. The XEN-P cells appear to be the principal self-renewing component of our rat XEN-P cell lines, since most newly emerging colonies consisted exclusively of Oct4/Gata6/Gata4/SSEA1-positive cells. These round, poorly adherent XEN-P cells did not express the extraembryonic endoderm markers SSEA3, Collagen 4, and Laminin B that were, however, up-regulated in older, heterogeneous colonies. The assertion that our cultures contain a precursor cell that can self-renew and differentiate into all extraembryonic endoderm cell types is further supported by our finding that single cell-derived sublines were morphologically and molecularly indistinguishable from the parent line. The normal pattern of colony evolution (Fig. 4) suggests that these clones derive from Oct4-positive cells, but it remains formally possible that some cells lose and then re-express Oct4 (see Fig. 1B and [29]). Collectively, the origin, molecular signature, differentiation, and developmental potential (including the ability to contribute to yolk sac endoderm in mice) of the rat XEN-P cells suggest strongly that they represent the committed extraembryonic endoderm precursor as thought to exist in mouse ICM [16]. History of mouse and rat ES cell isolation [30], [5], [6] suggests that study of the intracellular signaling pathways that regulate self-renewal and differentiation in the early extraembryonic endoderm may help to decide whether differences in the growth behavior of rat and mouse extraembryonic endoderm [31] are fundamental or not, and this strategy may eventually lead to the isolation of rat XEN and mouse XEN-P cell lines.

XEN-P cells may have unknowingly been isolated before. Indeed, a rat extraembryonic endoderm cell line was found to express SSEA1 [18], but since SSEA1 is not a strong marker per se, and since this cell line also expressed parietal endoderm markers (as our XEN-P cell lines do), other identities were not considered.

Although the XEN-P cells described here show the molecular signature and differentiation potential known or expected from the committed extraembryonic endoderm precursor, their path of origin remains to be understood. If these cell lines arise via a detour through an Oct4-negative stage (Fig. 1B), some plasticity may be involved. Two other observations pointing to plasticity in the early extraembryonic lineages include the apparent conversion of rat blastocyst-derived, Oct4^−^/Cdx2^+^ extraembryonic cell lines into extraembryonic endoderm cells [11] and a low contribution of XEN-P cell lines to the trophoblast [32]. Both of these observations require rigorous verification.

The discovery that permanently growing XEN-P cells express the *Oct4* gene is of great interest, especially when considering the close developmental relationship between nascent extraembryonic endoderm and nascent epiblast [16]. Oct4 is a central pluripotency factor [33]–[35], but can also trigger differentiation of ES cells [34], [36]. Until recently, it has not been seriously considered that the *Oct4* gene may be actively transcribed in the earliest committed step of the extraembryonic endoderm lineage. Indeed, although significant Oct4 protein levels have been detected in some or all cells of the primitive endoderm [37], [38; see however], [39], Oct4 mRNA levels decrease sharply in the primitive endoderm [e.g.], [37, 17]. Hence, during normal development, Oct4 protein in the primitive endoderm may be a product of earlier gene activity. However, it has recently been proposed that already before appearance of the primitive endoderm, the cells of the ICM become committed to either the epiblast or extraembryonic endoderm lineage [16], [17], [40; see], [however, 41]. Although all ICM cells contain Oct4 [15], it is not clear whether the *Oct4* gene would still be transcribed in any extraembryonic endoderm-committed ICM cells. Hence, the fact that our rat XEN-P cells transcribe the *Oct4* gene not only supports the argument that they represent the earliest known stage of the extraembryonic endoderm pathway, but also suggests that active *Oct4* gene expression (as opposed to carryover of Oct4 mRNA or protein from an uncommitted stage) is part of the gene expression profile defining that stage. Clearly, the *Oct4* gene expression in the XEN-P cells raises intriguing questions about the regulation and roles of Oct4 in the nascent extraembryonic endoderm, especially in light of the previous observation that forced overexpression of Oct4 in ES cells can cause their XEN-like differentiation [34].

The availability of permanently cultured cells that resemble the committed extraembryonic endoderm precursor opens interesting perspectives for the comparative analysis of extraembryonic endoderm precursor and epiblast precursor/ES cells, including whether Oct4 plays similar roles in these cells and how these roles are related to the fact that Oct4-deficient embryos cannot form an ICM [33]. Our new cell lines should also be useful for comparing extraembryonic endoderm precursor and epiblast precursor/ES cells regarding their epigenetic status and the signaling pathways involved in self-renewal [35], [42]. Like the Oct4, Rex1, and SSEA1 expression, our finding that LIF, an established mouse ES cell growth factor [42], stimulates the formation of extraembryonic endoderm cell colonies, suggests that substantial similarities are maintained between the epiblast and extraembryonic endoderm precursor populations. These comparisons will sharpen the molecular description of each cell type and in particular the molecular definition of pluripotency [35].

Finally, the rat XEN-P cell lines offer new possibilities for studying differentiation and function of the extraembryonic endoderm lineage. As a result of their high tendency to differentiate, the XEN-P cell lines constitute the first in vitro differentiation model in which extraembryonic endoderm cells are generated from their natural precursor cells. This contrasts with the traditional in vitro models where extraembryonic endoderm cells are generated in abnormal ways, i.e. from ES cells [43] (likely through a re-commitment - see discussions in references [16], [44]) or embryonic carcinoma cells [45]. Furthermore, by fractionating the XEN-P cell lines, it may become possible to reconstitute the visceral endoderm in order to study its developmental, physiological, and pathophysiological roles [46]–[50].

In conclusion, the cell lines presented here are an exciting new tool for examining the nature, differentiation, and plasticity of the committed extraembryonic endoderm precursor, new molecular functions of early embryonic regulators such as Oct4, and the development and biological roles of the extraembryonic endoderm.

An initial account of this work was presented in abstract form [51]. While this manuscript was being finalized, Li et al. [52] published a rat blastocyst-derived stem cell line that expresses Oct4 and Gata4. The differentiation potential of these cells was not tested, but unlike XEN-P cells, they grow on gelatin and in the absence of LIF. Clearly, it will be of interest to compare the two isolates.

## Materials and Methods

### Ethics statement

The animal experiments were approved by the Institutional Animal Care and Use Committee of Children's Memorial Research Center.

### Derivation and maintenance of XEN-P and XEN cell lines

Primary mouse and rat embryo fibroblasts (PMEFs and PREFs) were derived by standard methods [1]. For the derivation of rat XEN-P cell lines, blastocysts (4.5 days p.c.) were plated into Nunc 4-well dishes onto mitomycin C (10 µg/ml)-treated embryo feeders (detailed below) in DMEM (high-glucose, with glutamine and sodium pyruvate) supplemented with 0.1 mM beta mercaptoethanol, 15% fetal calf serum (ES-qualified), and 2,500 u/ml mouse LIF (ESGRO) at 37°C and 5% CO_2_. Rat XEN-P cell lines RX1 (strain WKY), RX2 (strain BDIX), and RX5 (WKY) were derived, respectively, on PREF, PMEF, and Li1; the latter is a permanent rat fibroblast feeder cell line that we obtained by spontaneous immortalization of PREFs prepared from a day-11 rat embryo (strain SD). 2 weeks after plating, the blastocyst outgrowths had completely converted into a XEN-like morphology. We pooled the primary outgrowths of 6–18 blastocysts, transferred the pools onto Li1, and each pool easily delivered the desired cell line. The rat XEN-P cell lines were maintained in the same medium used for derivation (except that LIF was reduced to 1,000 u/ml), and transferred every 2–3 days by trypsinization (0.25% trypsin-EDTA) onto mitomycin-treated Li1 cells (∼50,000 Li1 cells/cm^2^). The cells have been growing permanently without slowing down for >50 passages. The experiments described here were mostly performed between passages 30 and 40. At passages 39 and 40, line RX1 (the line used most for this study) contained 70–90% diploid and the rest tetraploid cells, as determined by flow cytometry after propidium iodide staining. Tetraploidy, which has been observed in previously isolated rat extraembryonic endoderm cells [18], does not reduce incorporation into extraembryonic tissues and has only a moderate effect on somatic incorporation before gastrulation [54], which is the time frame of the present study. Derivation of mouse XEN cell lines (strain NMRI) was analogous to that of rat XEN-P cell lines, but the XEN cell lines were maintained without LIF on PMEF feeders. D3 mouse ES cells [43] were maintained on mitomycin-treated PMEFs in the presence of 1000 u/ml LIF.

### Plating experiments

The trypsinized XEN-P cell lines or XEN-like primary outgrowths were filtered through a 40 µm Falcon strainer (which resulted in a mix of predominantly single cells and some 2-to-4-cell aggregates) and seeded into 4-well or 24-well plates at 100–500 (plating efficiency experiments with primary cells), 100–200 (plating efficiency experiments with cell lines) or 25–50 (immunocytochemistry experiments, cell lines only) cells per well onto mitomycin-treated Li1 cells (50,000–75,000 cells/cm^2^). At feeder densities ≤25,000/cm^2^, the fold increase in colony number caused by LIF became much greater than shown in Fig. 2B, but the plating efficiency was then extremely low (<1%) and colonies were only rudimentary, even with LIF (results not shown). To account for the substantial variations in plating efficiency (∼5–25% in presence of LIF and feeder) caused by variations in feeder cell density as well as by the variable degree of differentiation of the donor culture, the colony number in presence of LIF was set at 100%. Colonies were stained with crystal violet after 6–9 days, or they were stained with antibodies at the indicated time points.

### Reverse transcription (RT) PCR

Total RNA was isolated with the TRIZOL (Invitrogen) procedure or, for microsamples, by the RNeasy microkit (Qiagen). After treatment with DNase I, 2 µg (whole culture samples) or 50% (microsamples) of the RNA samples were reverse-transcribed with random hexamers using the first strand cDNA synthesis Superscript II kit from Invitrogen; control reactions excluded reverse transcriptase. Aliquots of the cDNA samples were subjected to regular PCR or real-time PCR, using an annealing temperature of 60°C (most experiments) or 62°C (Fig. 1B, C). Hprt was used as an internal standard that was co-amplified in the same tube (Fig. 1) or amplified in a separate reaction (Table 1). The real-time PCR was performed with the Platinum Quantitative PCR SuperMix (Invitrogen) in an ABI Prism 7700 sequence detection system (Applied Biosystems). The ratio of Oct4 mRNA levels of endoderm and ES cells was determined as 2 to the power of -deltadeltaCt, defined as the difference between the deltaCt (Ct[Oct4] minus Ct[hprt]) values of the samples of interest and the calibrator sample (ES cells). In order to ensure equal amplification efficiencies for rat and mouse targets, identical (“dual-specific”) primers were used. However, some of the Hprt primers were designed to distinguish mouse and rat in order to exclude cross-contaminations between rat and mouse samples. A list of primers is given in Table 3.

**Table 3:** List of primers used in this study.

Template cDNA	Forward primer (5′→3′); Reverse primer (5′→3′)	Product size (bp)	Mouse template (GeneBank)	Rat template (GeneBank)
Oct4 -	Ggcgttctctttggaaaggtgttc; actcgaaccacatccttctct	314	NM_013633	None
Oct4 -	Ggtggaggaagctgacaacaac; ggcaatgctagtgatctgctgc	172	None	XM_228354
Oct4	Gagggatggcatactgtggac; ggtgtaccccaaggtgatcc	272	NM_013633	XM_228354
Nanog	Tatcccagcatccattgcag; gtcctccccgaagttatggag	252	AB126939	AB162852
Sox2	Ccaagacgctcatgaagaagg; ctgatcatgtcccggaggtc	478	NM_011443	XM_574919
Fgf4	Tctactgcaacgtgggcatc; tggtccgcccgttcttac	285	NM_010202	NM_053809
Rex1	Tggagtacatgacaaaggggac; gcagccatcaaaaggacacac	509	NM_009556	XM_224882
Gata6	Gccgggagcaccagtaca; gtgacagttggcacaggacag	419	AF179425	NM_019185
Hnf4α	Gtgctgctcctaggcaatgac; cttgacgatggtggtgatgg	651	NM_008261	NM_022180
FoxA2	Agccccaacaagatgctgac; tggttgaaggcgtaatggtg	602	NM_010446	NM_012743
Ihh	Cctgtcagctgtaaagccagg; ggagcataggacccaaggg	336	NM_010544	AF175209
LamininB	Actacaccacgggccacaac; gcccaggtaattgcagacacac	440	NM_008482	XM_216679
Dab2	Ccacaggacaacctgcagtc; gccacagatgtggtaggacac	325	BC016887	NM_024159
Sparc	Attgcaaacatggcaaggtg; gccagtggacagggaagatg	474	NM_009242	NM_012656
Cdx2	Gcgaggactggaatggctac; tccttggctctgcggttc	499	NM_007673	NM_023963
Eomesodermin	Cggcaaagcggacaataac; gttgtcccggaagcctttg	361	NM_010136	AY457971
Placental lactogen	Ctgcttccatccatactccaga; gacaactcggcacctcaaga	410	XM_225307	NM_172156
Hprt -	Gcttgctggtgaaaaggacctct; ggaaatcgagagcttcagactcgtc	584	NM_013556	None
Hprt -	Gcttgctggtgaaaaggacctct; ccacaggactagaacgtctgctagttc	251	None	NM_012583
Hprt	Cagtcccagcgtcgtgattag; atccagcaggtcagcaaagaac	229	NM_013556	NM_012583

### Northern blotting

Northern blotting was performed using established procedures without significant modifications [55]. Total RNA was isolated with TRIZOL (Invitrogen), and 5 µg per lane were electrophoresed. The gels were blotted onto Hybond (Amersham) membranes, and hybridizations were performed using probes that were produced by RT-PCR and labeled with ^32^P-dCTP by the random priming procedure. The sources of the probe cDNAs were ES cells (Nanog) or XEN-P cells (Gata6 and Oct4), using the primers indicated in Table 3 for Nanog and Gata6 or primer pair ggagggatggcatactgtgg/accagggtctccgatttgc for Oct4.

### Western blotting

Western blotting was performed according to standard procedures [55] with minor modifications. Briefly, cells were dissolved in SDS-containing lysis buffer, protein was determined using Pierce's BCA kit, and the amounts indicated in the figure legends were separated on 10% or 4–20% SDS-PAGE gels, then electroblotted onto nitrocellulose or PVDF membranes. The membranes were blocked with TBS/Tween/5% milk powder and incubated with primary and secondary antibodies listed in Table 4.

**Table 4:** List of antibodies used in this study.

Antigen	Primary antibody and dilution*	Secondary antibody and dilution*
Oct4	SC-5279, 1∶100	SC-2068, 1∶1000; JI-115-036-003, 1∶10,000 (WB); SC-3699,1∶200; JI-115-486-003, 1∶200 (IF)
Oct4	SC-9081, 1∶200	SC-3842, 1∶300
Gata6	SC- 9055, 1∶200	SC-3842, 1∶300
Gata4	SC-9053, 1∶200	JI-111-036-003, 1∶10,000 (WB); JI-111-496-003, 1∶200 (IF)
Nanog	Ab-21603, 1∶200	SC-3842, 1∶300
Sox7	SC-20093, 1∶200	JI-111-036-003, 1∶10,000
Beta-Actin	A-2228, 1∶10,000	JI-115-036-003, 1∶10,000
SSEA-1	DSHB MC-480, 1∶500	SC-3699, 1∶200
SSEA-3	DSHB MC-631, 1∶100	I-81-6514, 1∶100
Laminin B2	DSHB D18, 1∶400	SC-3699, 1∶200
Collagen 4	DSHB M3F7, 1∶200	SC-3699, 1∶200

* SC, Santa Cruz Biotechnology, Inc.; Ab, Abcam plc; JI, Jackson Immunoresearch; DHSB, Developmental Studies Hybridoma Bank, University of Iowa.

### Transfection

For histochemical staining, the cells were seeded into 4-well dishes and transfected with 1 µg/well of plasmid GOF9 [53], using lipofectamine 2000 (Invitrogen). After 48 hours, beta-galactosidase activity was visualized by the method described in [55]. For production of clonal sublines, the cells were transfected in a 6-well plate with pSVneo (0.1 µg) and selected with G418 (200 µg/ml) for 2 weeks.

### Immunocytochemistry

Cells were seeded into Nunc 4-well plates at regular passaging density or at low density (up to 100 cells/well). At the time point of interest, immunocytochemistry was performed at room temperature. The wells were washed twice with PBS, fixed in 4% paraformaldehyde (10–15 minutes) and rinsed 3x with PBS. For intracellular antigens (Oct4, Gata6, Gata4), the cells were then permeabilized with 0.2% Triton X-100 in PBS (15–20 minutes) and rinsed 3x with PBS. Cells were blocked with 5% goat serum (Santa Cruz) in PBS (1 hour), incubated with primary antibodies overnight, rinsed 3x with 1% goat serum, and incubated with the secondary antibody conjugated to either FITC, TR or TRITC for 1 hour in the dark. For dual-color immunofluorescence, species-specific secondary antibodies were used. After secondary antibody incubation, the cells were washed 3x with 1% goat serum, incubated with 1 µg/ml of DAPI in PBS, and photographed under epifluorescence. The antibodies and their dilutions are listed in Table 4.

### Single cell FACS deposition and immunostaining

RX1 cells were single-cell deposited onto mitotically inactivated Li1 feeders in 96-well tissue culture plates using FACSAria II (BD Biosciences). Two weeks later, clones were stained with anti-Oct4 antibody (C-10; Santa Cruz) at a dilution of 1∶100 and visualized after staining with FITC-conjugated goat anti-mouse secondary antibody.

### Alkaline phosphatase cytochemistry

Alkaline phosphatase activity was visualized as described [55]. Briefly, cells were fixed with 4% paraformaldehyde (15 min), rinsed 3x with PBS, incubated for 30–60 minutes with staining solution (25 mM Tris-maleate [pH 9.0], 0.4 mg/ml a-naphthyl phosphate, 1 mg/ml Fast-Red TR salt, 8 mM MgCl_2_), rinsed again with PBS, and photographed.

### Labeling of XEN-P cell lines with GFP

Virus suspensions were produced using the EGFP-expressing lentiviral vector pFUGW and the packaging constructs pCMVΔR8.91 and pMD.G [56], which were generously provided by Dr. D. Baltimore (Caltech). The titers of virus stocks were determined as the percentage of EGFP-positive 293T cells transduced with serially diluted virus suspensions. For transduction, the XEN-P cell lines were seeded in 4-well plates (Nunc) at 5×10^4^ cells per well and incubated overnight. 2 hours before transduction, the medium was changed, and then transductions were carried out for 24 hours at an MOI of 1 in the presence of 8 µg/ml Polybrene (Sigma). The cells were then expanded, FACS-sorted to enrich the GFP-expressing fraction, and maintained for a few passages before injection.

### Microinjection experiments

All experiments were approved by the institutional review board. SD rats and CB 56 mice (Charles River, Wilmington, MA) were used to produce recipient embryos and pseudopregnant females. Cells were prepared for injection by trypsinization, sometimes in combination with collagenase treatment (1 mg/ml collagenase type IV, 15′), or by mechanical disaggregation with a Pasteur pipette. The cell samples (in their culture media) were then mixed with an equal volume of M2 and kept on ice until injection. Micromanipulations were performed with a Leica system as described [57], [58]. 3–5 cells were injected per rat blastocyst (E4.5) or mouse blastocyst (E3.5), followed by transfer into the uteri of pseudopregnant females. The conceptuses were dissected at the indicated times, and the labeled cells were located by fluorescence microscopy. Fluorescence images were acquired using a confocal Zeiss LSM 510 META Laser Scanning Microscope system (Thornmood, NY); the pinhole of the confocal microscope was partially opened in order to increase depth of focus while maintaining a good resolution.
